# The Influence of Li^+^ and K^+^ Added Cations and Annealing Temperature on the Magnetic and Dielectric Properties of Mg-Zn Ferrite

**DOI:** 10.3390/ma14174916

**Published:** 2021-08-29

**Authors:** Iulian Petrila, Florin Tudorache

**Affiliations:** 1 Faculty of Automatic Control and Computer Engineering, Gheorghe Asachi Technical University of Iasi, Boulevard Dimitrie Mangeron, No. 67, 700050 Iasi, Romania; iulianpetrila@yahoo.com; 2 Institute of Interdisciplinary Research, Department of Exact and Natural Sciences, Ramtech Center, Alexandru Ioan Cuza University of Iasi, Boulevard Carol I, No. 11, 700506 Iasi, Romania

**Keywords:** magnesium-zinc ferrite, hysteresis loops, dielectric permittivity, coercivity

## Abstract

This paper presents the results of an investigation on the magnetic and dielectric properties of Mg_0.5_Zn_0.5_Fe_2_O_4_ spinel ferrite with a 1% weight percentage of Li^+^ and K^+^ added cations. The addition of metal ions plays an important role in increasing the porosity and favors the formation of ferrite at low temperatures. The goal of this new research is to demonstrate that by selecting the type of metallic cations for addition or choosing an optimal sintering temperature, it may be possible to improve the magnetic and electrical properties of Mg-Zn ferrite. The samples were prepared using sol-gel self-combustion techniques and annealed at 1000 °C, 1100 °C, and 1200 °C. Scanning electron microscopy revealed the shape and grain size of the samples, and the phase composition was analyzed using the X-ray diffraction technique. The magnetic information, such as remanent magnetization M_R_, saturation magnetization M_S,_ and coercivity H_C_, were extracted from the hysteresis loops of the samples. The electrical investigation was focused on the low- and high-frequency dependence of dielectric constant and dielectric losses. The results are discussed in terms of microstructural changes induced by the additions of Li^+^ and K^+^ metallic cations. Conclusions are drawn concerning the optimization of magnetic and electrical properties for the development of Mg-Zn ferrite with possible applications in the field of magnetic materials or electronics.

## 1. Introduction

Ferrites based on Mg-Zn are important ceramic materials that present spinel-type lattices with soft magnetic properties.There isenhanced interest in these materials due to their chemical stability and low production costs for possible applications such as magnetic recording, electromagnets, hyperthermia treatment, antibacterial activity, humidity sensors, medicine, and inductive sensors and actuators working in a wide frequency range 10^2^ to 10^9^ Hz [1,2,3,4,5,6].

The main inconvenience of spinel magnesium-based ceramic materials is their high annealing temperature, over 1300 °C, which causes significant material loss and large energy consumption. Magnetic and dielectric properties of ferrites can be enhanced or altered by modifying various added metallic cations and the sintering temperature and/or time [7,8,9,10,11]. Magnetic and dielectric properties of spinel ferrites AB_2_O_4_ are also dependent on the distribution of A and B cations in tetrahedral or octahedral sites, respectively. The various cationic distributions were studied by different authors and reported in various papers [12,13,14,15,16]. The common conclusion of these works was that the temperature of synthesis influences the distribution of metal cations in the position of the spinel network. The Mg^2+^ cations are usually placed on octahedral sites, and only a minority are placed in tetrahedral sites of sublattice [17,18].

The main goal of this work is to investigate the evolution of the magnetic and dielectric properties of magnesium-zinc ferrite as a result of the addition of two alkaline metallic cations with the same valences Li^+^ and K^+^. The addition of alkaline metal cations of Li^+^ and K^+^ to the base structure of the magnesium-zinc ferrite was chosen because alkaline metals present a high reactivity and special physico-chemical properties, which can easily integrate into the structure of ferrite. Because alkaline metals are very reactive, they have a single electron on the last layer, which they readily give up to other metals in the ferrite composition. The addition of metal cations to the magnesium-zinc ferrite structure can make an essential contribution to improvingthe conduction mechanism, optical or magnetic properties of semiconductors [19,20,21].

Magnetic and dielectric properties at room temperatures for Mg-Zn ferrites were reported by various authors [22,23,24,25]. High dielectric permittivity values can be reached using oxides of transition elements, and this is the main reason for the synthesis of Mg-Zn ferrites with Li^+^ and K^+^ added cations. Controlling the microstructure and selecting the intrinsic resistivity are the most important factors for achieving good values of permittivity, above ε_r_ > 1000.

Therefore, the preparation technique and the sintering conditions of the samples were considered to be very important. We selected Mg-Zn ferrite for the following reasons: it is a lighter ceramic material, and the stability of the Mg^2+^ ion avoids the appearance of Fe^2+^ ions (an essential requirement to obtain good values of permittivity); magnesium-zinc ferrite has the largest application field including, magnetic materials, electronics, or sensors; it is relatively easy to prepare, and all the materials involved in the preparation of ferrite are friendly for the environment.

## 2. Materials and Methods

Various preparation methods were used to obtain Mg-Zn ferrite, such as sol–gel, sol-gel auto-combustion, co-precipitation, and thermal evaporation [26,27,28,29,30]. Reference samples with stoichiometric composition Mg_0.5_Zn_0.5_Fe_2_O_4_ were prepared in an air atmosphere by using the sol-gel self-combustion method [31]. For the preparation of ferrite by the sol-gel self-combustion method, we have chosen the nitrate because ammonia and polyvinyl alcohol can form, in some ratios and in the dry state, pyrotechnic mixtures in which ammonia is the oxidant, and polyvinyl alcohol is combustible. Such a mixture, once ignited, undergoes an exothermic and self-sustaining reaction that generates a sufficient amount of heat to eliminate the water. Calculations were carried out to obtain 0.05 moles of ferrite Mg_0.5_Zn_0.5_Fe_2_O_4_ resulting in the following amounts of nitrates: Mg(NO_3_)_2_·6H_2_O = 6.075 cm^3^; Zn(NO_3_)_2_·6H_2_O = 16.34 cm^3^; Fe(NO_3_)_3_·9H_2_O = 55.8 cm^3^, and polyvinyl alcohol = 80.61 cm^3^. Required quantities of high-grade purity precursors Mg(NO_3_)_2_·6H_2_O; Zn(NO_3_)_2_·6H_2_O; Fe(NO_3_)_3_·9H_2_O and polyvinyl alcohol [CH_2_CHOH]_n_ from Sigma–Aldrich, was weighed according to the stoichiometric formula of desired final composition and dissolved in small amounts of distilled water. Polyvinyl alcohol solution was added in molar ratio 1:1 to make a colloidal solution. The constituents were mixed and neutralized (pH = 7) with solution 10% ammonia (NH_4_OH) concentration. To initiate an ignition combustion reaction, each resulting mixture composition was dried for 4 h at 100 °C and, after combustion, resulted in Mg_0.5_Zn_0.5_Fe_2_O_4_ ferrite powder.

Equal amounts of Li_2_CO_3_ = 1.0221g and K_2_CO_3_ = 1.0221g powders as a source of Li^+^ and K^+^ cations were added and mixed with the magnesium-zinc ferrite composition.

Since the self-combustion reaction cannot be complete in the whole mass of material, a primary pre-calcining treatment wasrequired, which was carried out for 1 h at 500 °C in an air atmosphere. Desired shapes of specimens (6 mm diameter and 1–2 mm thickness pellets) were obtained by uniaxially pressing the pre-calcined powder at 5 × 10^7^ N/m^2^ using a Carver model 4350 hydraulic press. In order to obtain the best homogeneity of the mixture between the ferrite and the addition of metal cations, the heat treatment was carried out for 4 h.

In order to observe the influence of annealing temperature on structural, magnetic, and dielectric properties of compacts specimens, three different temperatures were chosen for the thermal treatment: 1000 °C, 1100 °C, and 1200 °C, respectively, for 4 h. The internal mechanical stresses that can occur by thermal shocks were avoided using a slow sample cooling process at the end of each treatment. A mechanical polishing ensured the planarity and the parallelism of the faces of the sample and deposition of thin silver contact electrodes on both faces.

The phase components analysis was performed at room temperature using a Shimadzu LabX-6000 X-ray diffractometer with Bragg-Brentano focusing system and CuKα radiation (α = 1.5405 Å) source.

The SEM images were obtained using a scanning electron microscope model Quanta 200 on freshly fractured samples. The investigation was focused on crystallite agglomeration tendencies and crystallites’ mean size. The roughness of the surface was performed using the 3D optical surface profilometer model ZygoZeGage.

The investigations of magnetic properties were performed on a vibrating sample magnetometer (VSM) at room temperature. For these measurements, the 4 mm diameter spherical samples were fabricated by mechanical polishing. The maximum intensity of the magnetic field was 900 kA/m.

The dependence of electrical permittivity on the frequency was recorded using a Wayne Kerr Impedance Phase Analyzer 6400P in the range of 1 Hz–1 MHz.

## 3. Results and Discussion

The effect of the addition of Li^+^ and K^+^ cations in the Mg-Zn ferrite is discussed in terms of sintering behavior, structure evolution, magnetical properties improvement, and dielectric characterization of these materials.

### 3.1. Microstructural Characterization

**Figure 1:**
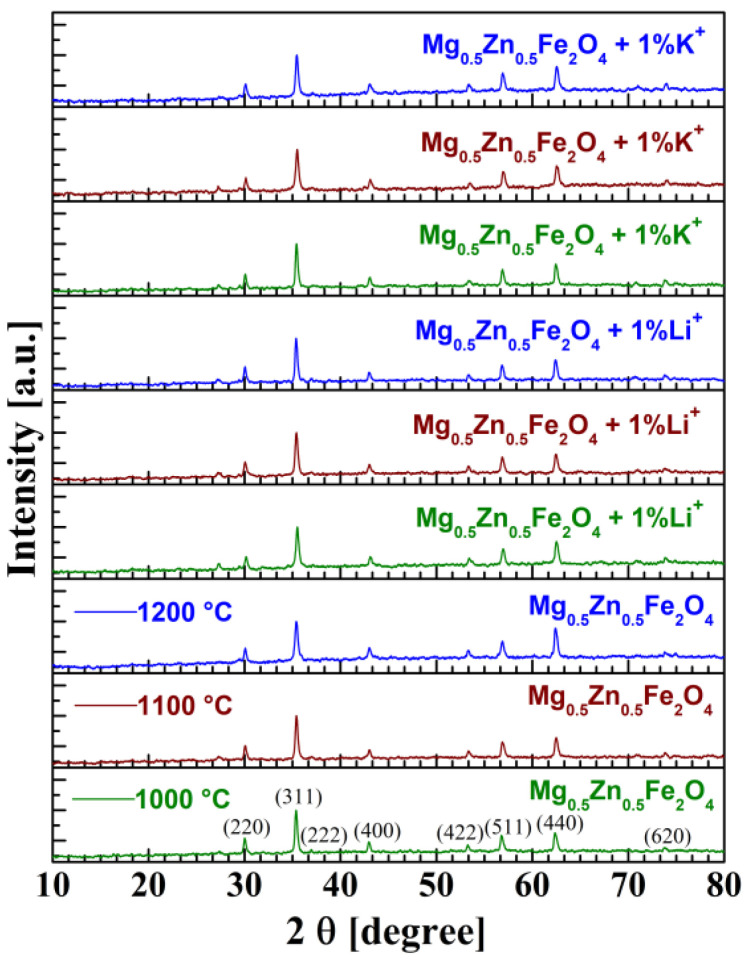
XRD patterns of magnesium-zinc ferrites.

Comparative analysis of recorded X-ray diffractograms (Figure 1) emphasized the spinel structure—cubic phase of each sample: reference, Li^+^ and K^+^ samples. Diffraction lines belonging to the spinel phase are identified, the highest intensity peak (311) is present at 2θ = 36°, and all diffractograms have confirmed the single-phase character of samples, similar to ones reported in the literature [32,33,34,35].

We also observed that increasing the annealing temperature allowed the decrease of the porosity of the samples. From the geometrical dimensions and the weight of each sample, we present in Table 1 the calculated porosity values of Mg-Zn ferrites. A slight increase in the porosity of the samples with cations additions in comparison with the basic ferrite was observed. In the case of Mg-Zn ferrite sintered at 1000 °C, the addition of Li^+^ cations caused an increase in porosity of 3%, and the addition of K^+^ cations caused an increase in porosity of 9%. A significant increase in porosity wasobserved for ferrites sintered at 1200 °C, where the addition of Li^+^ cations caused an increase in porosity of 20% and the addition of K^+^ cations caused an increase in porosity of 35%. The increase can be explained bases on the atomic radius of the substituent cations. Similar results about the evolution of porosity were reported in the literature by [32].

**Figure 2:**
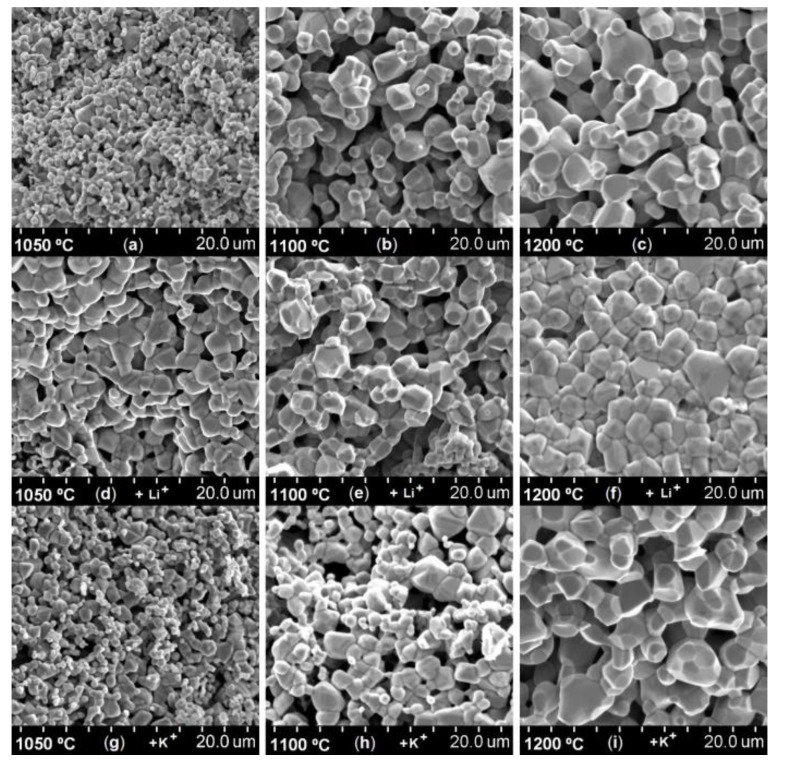
SEM images of the magnesium-zinc ferrites before and after thermal treatment (t = 4h) and cations addition (**a**) T = 1000 °C; (**b**) T = 1100 °C; (**c**) T = 1200 °C; (**d**) 1% Li^+^, T=1000 °C; (**e**) 1% Li^+^, T = 1100 °C; (**f**) 1% Li^+^, T = 1200 °C; (**g**) 1% K^+^, T = 1000 °C; (**h**) 1% K^+^, T = 1100 °C; (**i**) 1% K^+^, T = 1200 °C.

The microstructure investigations can explain the evolution of the porosity for each sample. The SEM images of fractures of 1000 °C, 1100 °C, and 1200 °C sintered samples are presented in Figure 2. These show the shape and the dimensions of crystallites and the absence of the secondaryphase. For the samples containing Mg_0.5_Zn_0.5_Fe_2_O_4_ + 1%Li^+^, the increase of crystallites dimension is inhibited by the lithium cations segregation.

Moreover, we observed the influence of K^+^, which alloweda fine-grained structure of the sample, and that the granulation can be increased by increasing the annealing temperature.

**Figure 3:**
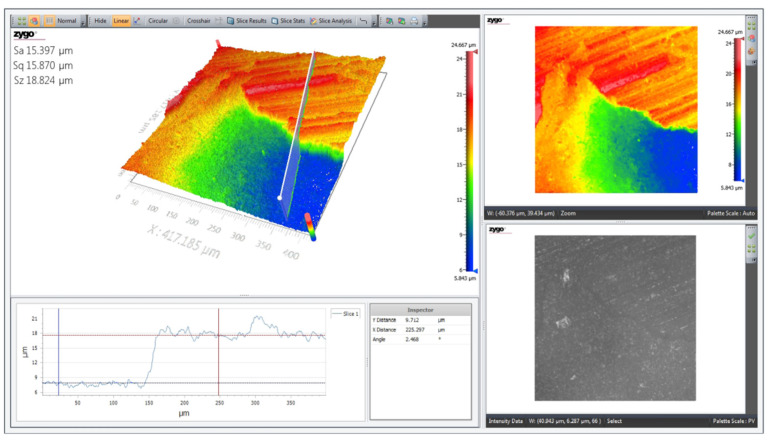
The 3D topographic surface profile of Mg_0.5_Zn_0.5_Fe_2_O_4_ ferrite sintered at 1100 °C.

The 3D topographic profile of the surface of the Mg-Zn ferrite sample was made using a non-contact optical profilometer model ZygoZeGage at room temperature and pressure. Figure 3 shows the representative parameters of the calculated sample area: R_a_—represents the arithmetic area; S_a_—represents the mathematical area of the pixels; S_q_—represents the square area of the pixels, and S_z_—represents the topographic difference between the highest and lowest point. Analyzing the surface topography of the ferrite sample shown in Figure 3, we noticed that the surface is uniform and, in some places, has present agglomerations of crystallites, which is in agreement with the SEM micrographs shown in Figure 2.

### 3.2. Magnetic Properties

It is a well-known fact that the magnetization of ferrites depends on factors such as the addition or substitution of different cations in the ferrite composition, iron cations distributions between the A and B sites of the spinel structure, inhomogeneities, or crystallites size [17,36].

We found that the magnetic properties are influenced by the type of the additions and the annealing temperature. The specific saturation magnetization M_s_ was measured with a vibrating sample magnetometer in a magnetic field range −900–900 kA/m using spheres prepared from the pellet samples.

**Figure 4:**
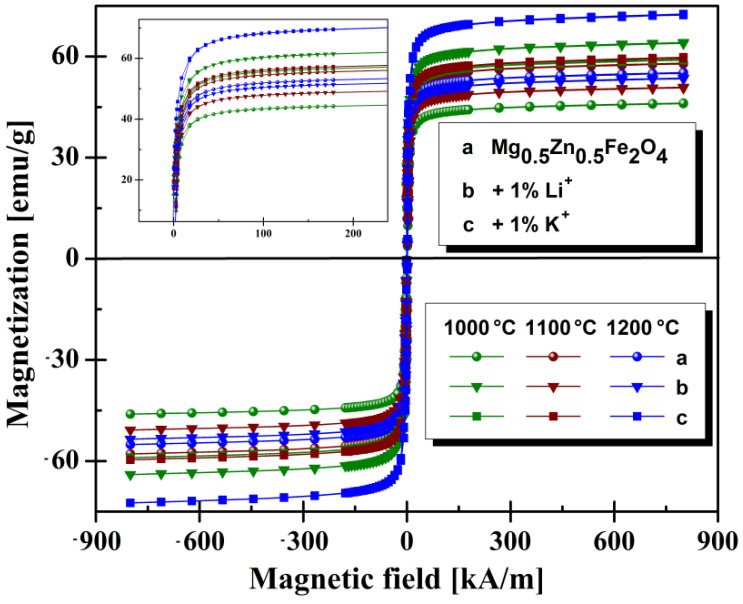
Magnetic hysteresis loops M (H) at room temperature obtained for investigated magnesium-zinc ferrites.

The investigation of magnetic properties confirmed that Mg-Zn ferrites are soft magnetic materials with high magnetic permeability and near to zero values for coercivity H_c_ (Figure 4).That is in concordance with results reported in the literature [21,32,37,38,39,40].

For the reference composition Mg_0.5_Zn_0.5_Fe_2_O_4_, sintered at 1000 °C for 4 h, the saturation value is high (45 emu/g), and remanence values are low (1.9 emu/g), as shown in Figure 4. For the same composition, changing the annealing temperature to 1100 °C results in the remanence having a significant increase to 2.7 emu/g (almost 45%), and the saturation rises to 56.8 emu/g. At a higher annealing temperature (1200 °C) used in the sample preparation, the remanent magnetization decreases to 1.5 emu/g, accompanied by a lowering of the saturation. Therefore, for the reference Mg_0.5_Zn_0.5_Fe_2_O_4_ ferrite, the optimal annealing temperature for increasing both M_r_ and M_s_ is 1100 °C.

In the case of ferrites having Li^+^ cations included in the composition and sintered at 1000 °C, the measured value for saturation magnetization was 62.9 emu/g, and remanence magnetization was 2.8 emu/g. Relative to the reference sample, M_s_ and M_r_ were increased in the presence of Li^+^ cations (Figure 4).

The change in annealing temperature from 1000 °C to 1100 °C led to a decrease inmagnetic properties. The explanation for this decrease is linked to the action of Li^+^ cations upon grain size, in the sense of their diminishing. The annealing temperature of 1200 °C slightly increasedthe magnetic parameters at values lower than those of 1000 °C but comparable with values of the reference sample at the same temperature.

Magnetic properties of Mg_0.5_Zn_0.5_Fe_2_O_4_ with K^+^ cations (Figure 4), sintered at 1000 °C, are comparable to ferrites with Li^+^ cations, and therefore higher than the reference sample. Comparing the SEM images of sample structure, the change in granulation is one possible explanation. Grains of ferrites with K^+^ cations, obtained at 1200 °C, are much more coarse and faceted compared to the reference sample (Figure 2c).

The relevant magnetic properties extracted from hysteresis measurement are summarized in Table 2. We found that the addition of metal cations to the composition of magnesium-zinc ferrite involved a significant increase of coercivity for all samples.

As can be seen in Table 2, the intermediate temperature values are comparable with reference, but a spectacular increase is observed at 1200 °C, where the value of M_s_ is 72.3 emu/g, and M_r_ is 3.1 emu/g. The variations of the magnetic parameters may be ascribed to compositional perturbations produced by the presence of atoms Li^+^ or K^+^ in the host spinel lattice. This results in a magnetic dilution of the octahedral sublattice. A significant increase of saturation is observed for ferrites sintered at 1000 °C, where the addition of Li^+^ cations causes an increase of saturation by over 39%, and for ferrites sintered at 1200 °C, the addition of K^+^ cations causes an increase of saturation by over 31%. Comparing our magnetic properties with the results reported by T. Tatarchuk [21], it is found that the addition of Li^+^ and K^+^ cations to the Mg-Zn spinel ferrite implies the obtaining of superior magnetic properties. Thus, the increase of values in the case of saturation (M_s_) and coercivity (H_c_) is observed.

### 3.3. Dielectric Properties

For applications in the field of electronics, the dependence on the electrical permittivity of the frequency is very important. In the case of spinel ferrites, the number of Fe^2+^ ions placed in octahedral sites of lattices plays an important role in the conduction mechanism. The electronic conduction mechanism is based on electron exchanges between ions of the same species that are randomly distributed and have different valence states.

**Figure 5:**
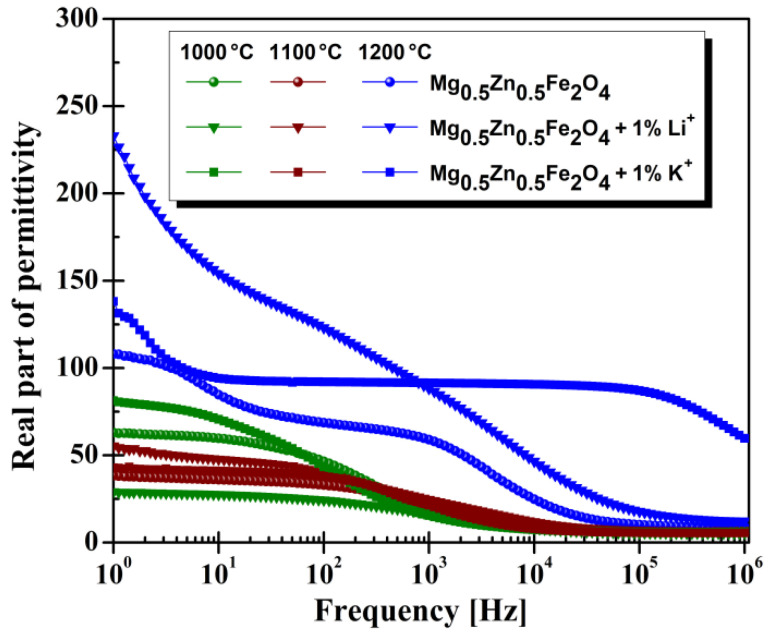
Dielectric characteristics of the Mg-Zn ferrites.

The measurements on the frequency dependence of electrical permittivity are presented in Figure 5. We found that the compositional and annealing temperature changes also influenced the electrical permittivity. High values at low frequency for the real part of permittivity confirm the semiconducting characteristic of this category of materials. These characteristics were also reported in the literature [41,42]. The remarkable case is, in our investigation, the Mg-Zn ferrites with K^+^ cations. For 1100 °C annealed samples, the real permittivity value is nearly constant in the range of frequencies 1–100 Hz. Increasing the sintering temperature to 1200 °C, this constant interval is extended to 10^5^ Hz (Figure 5).

**Figure 6:**
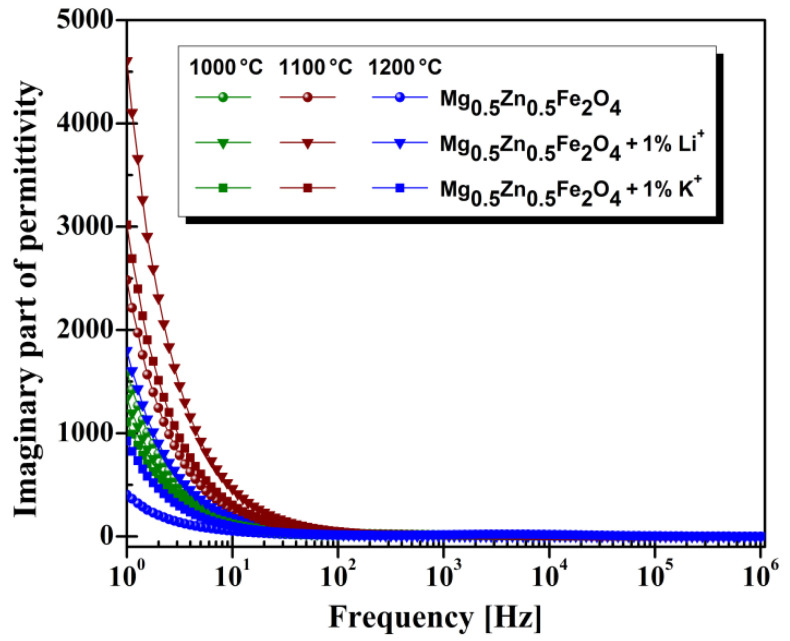
Imaginary part of permittivity vs. frequency for investigated magnesium-zinc ferrites.

For all samples, the imaginary part of permittivity exponentially decreases in the range 1–10^6^ Hz, with a quick tendencyat low frequencies, 1–10 Hz, and a slow one from 10^2^ to 10^6^ Hz (Figure 6). Similar results were reported in the literature by [43,44].

A Wayne Kerr Impedance Phase Analyzer was used to investigate the variation of the electric permittivity in the frequency range 10^7^ ÷ 10^9^ Hz.

**Figure 7:**
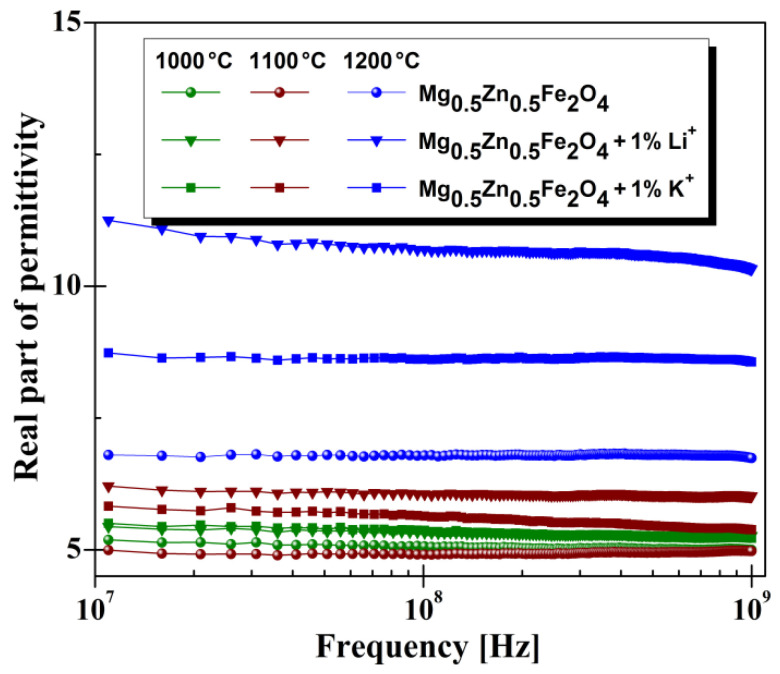
High-frequency characteristics of real part of permittivity for investigated Mg-Zn ferrite.

**Figure 8:**
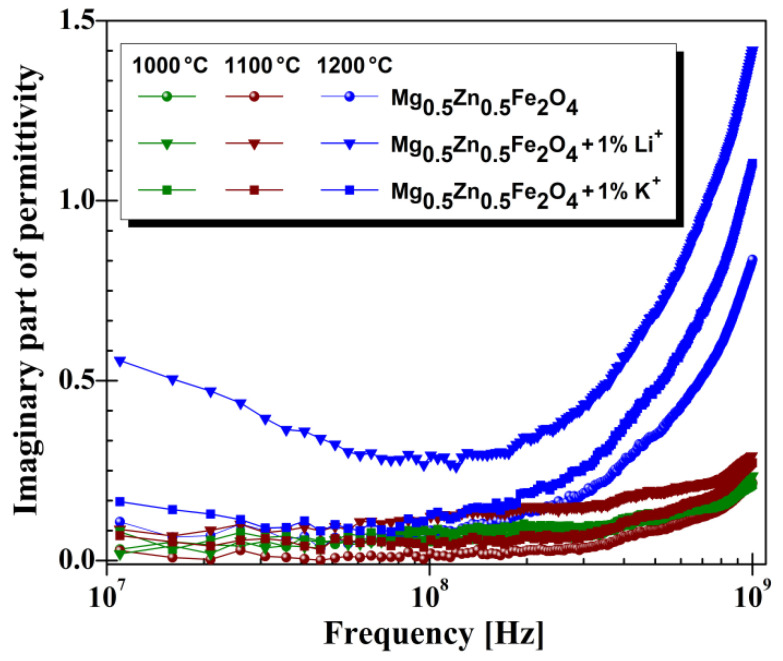
High-frequency characteristics of imaginary part of permittivity for investigated Mg-Zn ferrites.

As can be seenin Figure 7, high frequency, unlike low frequency, samples treated at high temperatures showed high values of permittivity. As shown in Figure 7, in the range 10^7^–10^9^ Hz, the real part of permittivity (*ε′*) remains constant for all analyzed samples, which means good stability to a higher frequency. Similar behavior of dielectric constant was also reported in the literature [19]. Comparing our dielectric properties with the results reported by M.A.Rahman [19] found that the addition of Li^+^ and K^+^ cations to Mg-Zn spinel ferrite obtained superior dielectric properties.Thus, a doubling of the values of the dielectric constant for the high-frequency domain was observed. Also, it was observed that added cations in the Mg-Zn ferrite increased the real part of permittivity; the highest values were observed for Li^+^ added cations, irrespective of temperature treatment. The same rules can also be observed in the case of the imaginary part of permittivity (see Figure 8).

The influence of temperature treatment on the dielectric losses can be observed for all samples at very high frequency, in that high-temperature treatment increases the value of imaginary part of permittivity (*ε′′*) near to 10^9^ Hz.

## 4. Conclusions

In this paper, we show that porous Mg-Zn ferrite can be obtained at medium temperatures using the addition of alkaline metal cations. The different behaviors of the samples are caused by the annealing temperature and different microstructures in close correlation with the action of the cations additions.

As a result of studies, we found that the physical parameters of Mg-Zn ferrite are more or less influenced by the presence of foreign cations introduced as additives to the structure of ferrite. The study of the Mg-Zn ferrite with spinel structure was determined by the necessity to obtain good porosity, superior magnetic or electrical properties by performing controlled additions of Li^+^ and K^+^ cations to the host composition of the ferrite.

The annealing temperature and type of added cations play an important role in the modification of the microstructure, magnetic, and dielectric properties.

We found that the porosity of all samples decreases with increased annealing temperature due to the increase of particle grain size. Significant increases in porosity are observed for samples sintered at 1200 °C, where the addition of Li+ cations involves an increase of porosity by 20%, and the addition of K+ cations involves an increase of porosity by 35% compared with the reference sample.

Significant increases of saturation are observed for ferrites sintered at 1000 °C, where the addition of Li^+^ cations causes an increase of saturation by over 39%, and for ferrites sintered at 1200 °C, the addition of K^+^ cations causes an increase of saturation by over 31%.

Another important result is the constant value for dielectric permittivity in the frequency range of 10–10^5^ Hz for Mg_0.5_Zn_0.5_Fe_2_O_4_ ferrites with the addition of K^+^, annealed at 1200 °C.

We demonstrated that by selecting an appropriate cationic addition or by choosing an optimal sintering temperature, it might be possible to improve the magnetic and electrical properties of Mg-Zn ferrite.

## Figures and Tables

**Table 1:** The porosity of Mg-Zn ferrites.

Sample	Annealing Temperature [°C]	Porosity [%]
Mg_0.5_Zn_0.5_Fe_2_O_4_	1000	32
Mg_0.5_Zn_0.5_Fe_2_O_4_	1100	29
Mg_0.5_Zn_0.5_Fe_2_O_4_	1200	20
Mg_0.5_Zn_0.5_Fe_2_O_4_ + 1%Li^+^	1000	33
Mg_0.5_Zn_0.5_Fe_2_O_4_ + 1%Li^+^	1100	31
Mg_0.5_Zn_0.5_Fe_2_O_4_ + 1%Li^+^	1200	24
Mg_0.5_Zn_0.5_Fe_2_O_4_ + 1%K^+^	1000	35
Mg_0.5_Zn_0.5_Fe_2_O_4_ + 1%K^+^	1100	31
Mg_0.5_Zn_0.5_Fe_2_O_4_ + 1%K^+^	1200	27

**Table 2:** Summary of the magnetic properties of Mg-Zn ferrites.

Sample	Annealing Temperature [°C]	Saturation M_s_ [emu/g]	Remanence M_R_ [emu/g]	Coercivity H_C_ [kA/m]
Mg_0.5_Zn_0.5_Fe_2_O_4_	1000	45.0	1.9	1.1
Mg_0.5_Zn_0.5_Fe_2_O_4_	1100	56.8	2.7	1.3
Mg_0.5_Zn_0.5_Fe_2_O_4_	1200	55.0	1.5	1.2
Mg_0.5_Zn_0.5_Fe_2_O_4_ + 1%Li^+^	1000	62.9	2.8	7.1
Mg_0.5_Zn_0.5_Fe_2_O_4_ + 1%Li^+^	1100	49.7	1.3	5.7
Mg_0.5_Zn_0.5_Fe_2_O_4_ + 1%Li^+^	1200	52.4	1.5	6.2
Mg_0.5_Zn_0.5_Fe_2_O_4_ + 1%K^+^	1000	57.9	2.2	8.7
Mg_0.5_Zn_0.5_Fe_2_O_4_ + 1%K^+^	1100	59.5	2.5	8.9
Mg_0.5_Zn_0.5_Fe_2_O_4_ + 1%K^+^	1200	72.3	3.1	9.2

## Data Availability

Exclude this statement.
